# Neonicotinoid residues in UK honey despite European Union moratorium

**DOI:** 10.1371/journal.pone.0189681

**Published:** 2018-01-03

**Authors:** Ben A. Woodcock, Lucy Ridding, Stephen N. Freeman, M. Gloria Pereira, Darren Sleep, John Redhead, David Aston, Norman L. Carreck, Richard F. Shore, James M. Bullock, Matthew S. Heard, Richard F. Pywell

**Affiliations:** 1 NERC Centre for Ecology & Hydrology, Wallingford, Oxfordshire, United Kingdom; 2 NERC Centre for Ecology & Hydrology, Lancaster Environment Centre, Bailrigg, Lancaster, United Kingdom; 3 BBKA Technical and Environmental Committee, The British Beekeepers Association, The National Beekeeping Centre, National Agricultural Centre, Stoneleigh Park, Warwickshire, United Kingdom; 4 Laboratory of Apiculture and Social Insects, School of Life Sciences, University of Sussex, Falmer, Brighton, East Sussex; University of California San Diego, UNITED STATES

## Abstract

Due to concerns over negative impacts on insect pollinators, the European Union has implemented a moratorium on the use of three neonicotinoid pesticide seed dressings for mass-flowering crops. We assessed the effectiveness of this policy in reducing the exposure risk to honeybees by collecting 130 samples of honey from bee keepers across the UK before (2014: N = 21) and after the moratorium was in effect (2015: N = 109). Neonicotinoids were present in about half of the honey samples taken before the moratorium, and they were present in over a fifth of honey samples following the moratorium. Clothianidin was the most frequently detected neonicotinoid. Neonicotinoid concentrations declined from May to September in the year following the ban. However, the majority of post-moratorium neonicotinoid residues were from honey harvested early in the year, coinciding with oilseed rape flowering. Neonicotinoid concentrations were correlated with the area of oilseed rape surrounding the hive location. These results suggest mass flowering crops may contain neonicotinoid residues where they have been grown on soils contaminated by previously seed treated crops. This may include winter seed treatments applied to cereals that are currently exempt from EU restrictions. Although concentrations of neonicotinoids were low (<2.0 ng g^-1^), and posed no risk to human health, they may represent a continued risk to honeybees through long-term chronic exposure.

## Introduction

Neonicotinoid pesticides are the most widely used class of insecticides and account for around one third of the worldwide market [1]. They are most commonly applied as prophylactic seed coatings on a wide variety of flowering (e.g. oilseed rape and sunflower) and non-flowering (e.g. wheat and maize) crops [2, 3]. Their systemic expression in the tissues of plants provides targeted protection against herbivorous pests, including those that show resistance to previously developed pesticides, such as pyrethroids [2, 3]. However, their systemic nature also means that neonicotinoids are found in the pollen and nectar of mass-flowering crops attractive to pollinating insects, including honeybees and wild bees [4, 5]. A considerable body of recent research has linked this route of exposure to negative impacts on insect pollinators [4, 6–9]. These concerns have resulted in a European Union (EU) temporary moratorium on the application of three classes of neonicotinoid seed treatments–imidacloprid, clothianidin and thiamethoxam [10]. This restriction in the EU is for mass flowering crops (principally oilseed rape and sunflower) and does not include winter sown cereals. This moratorium came into effect for crops sown in the spring of 2014 and is still in place [10–12] (Table A in S1 Appendix).

Typically 2–20% of neonicotinoid seed coatings are absorbed into the germinating crop, which leaves a potentially large proportion of the pesticides as residues in the soil [13]. Soil degradation times of neonicotinoids are also highly variable, with *DT*_*50*_ (soil half-life) values ranging from 200 to over 1000 days for clothianidin, thiamethoxam and imidacloprid [5]. Both of these factors are likely to contribute to the identification of soil contamination following the use of neonicotinoids on crops. For example, under typical field conditions concentrations as high as 13.6 μg kg^-1^ were reported in arable soils before the moratorium [14]. There is considerable potential for neonicotinoids to persist in soils, even after cessation of their use on mass flowering crops [5, 13]. Where this occurs they may continue to be found in mass flowering crops grown on the same soils, thus posing a risk of exposure for bees feeding on their pollen and nectar [5]. A further exposure pathway for pollinators is through drift of dust or movement in surface water from cereal crops into field edges which can result in neonicotinoid residues being found in wild flowers and shrubs growing in these areas [5, 15–18].

Despite the EU moratorium on neonicotinoid seed dressing use in mass flowering crops, there has been no systematic monitoring to determine how effective this has been in reducing exposure risk to insect pollinators. We report the findings of a national survey of neonicotinoid residues found in honey collected in Great Britain. Honey samples were sourced from amateur beekeepers both before (2014) and after (2015) the implementation of the EU moratorium on neonicotinoid use. The residues in honey were then related to the areas of both oilseed rape, winter sown cereals and total arable cover that surrounded the sampled apiaries. We hypothesized that: 1) residues of neonicotinoids would be detected in honey stored by honeybees at higher rates before the moratorium that after it; 2) following the moratorium residues of neonicotinoids would be detected in honey as a result of bees foraging on flowering plants grown on soils containing persisting neonicotinoid residues; 3) where these residues persisted the most likely mechanism of exposure would be untreated mass flowering crops (oilseed rape) sown into soils contaminated by previous neonicotinoid use [14, 15]. In line with hypothesis 3), we predicted that despite the moratorium, residues of neonicotinoids in honey would remain correlated with the area of oilseed rape (the principal mass flowering crop grown in the UK) around each apiary.

## Materials and methods

### Honey samples

The use of stored hive products (pollen, nectar or honey) has been used to assess exposure risks to pesticides for honeybees [19–23]. This including a worldwide survey of neonicotinoid residues [24]. Here we expand on this previous research by using similar approaches to quantify neonicotinoid persistence following a ban on its use in mass flowering crops and linking this exposure to local cropping patterns. Although large commercial apiaries produce the majority of consumed honey in the UK, there are *c*. 24,000 amateur apiarists. Typically amateur apiarists keep only one to five hives during a season with honey production for either personal consumption or small scale commerce. Honey samples were solicited from these amateur apiarists from 23/2/2016 until 20/5/2016 through a request advertised in the newsletter of The British Beekeepers Association as well as the “The Scottish Beekeeper” magazine. The provision of honey samples was on an entirely voluntary basis and resulted in 122 apiarists providing samples from one or more years from 2014 (N = 23), 2015 (N = 147). In all cases postage costs were covered, although no direct payment was made to beekeepers. It should be noted that the study was not designed to make inferences about the decision making process for why beekeepers provided us with samples, which were likely specific to each individual. The impact of such factors was beyond the scope of the current study, however, there is no *a priori* reason to expect this to influence the results. The voluntary provision of honey samples have been used to assess pesticide residues in previous studies [22, 24].

**Fig 1:**
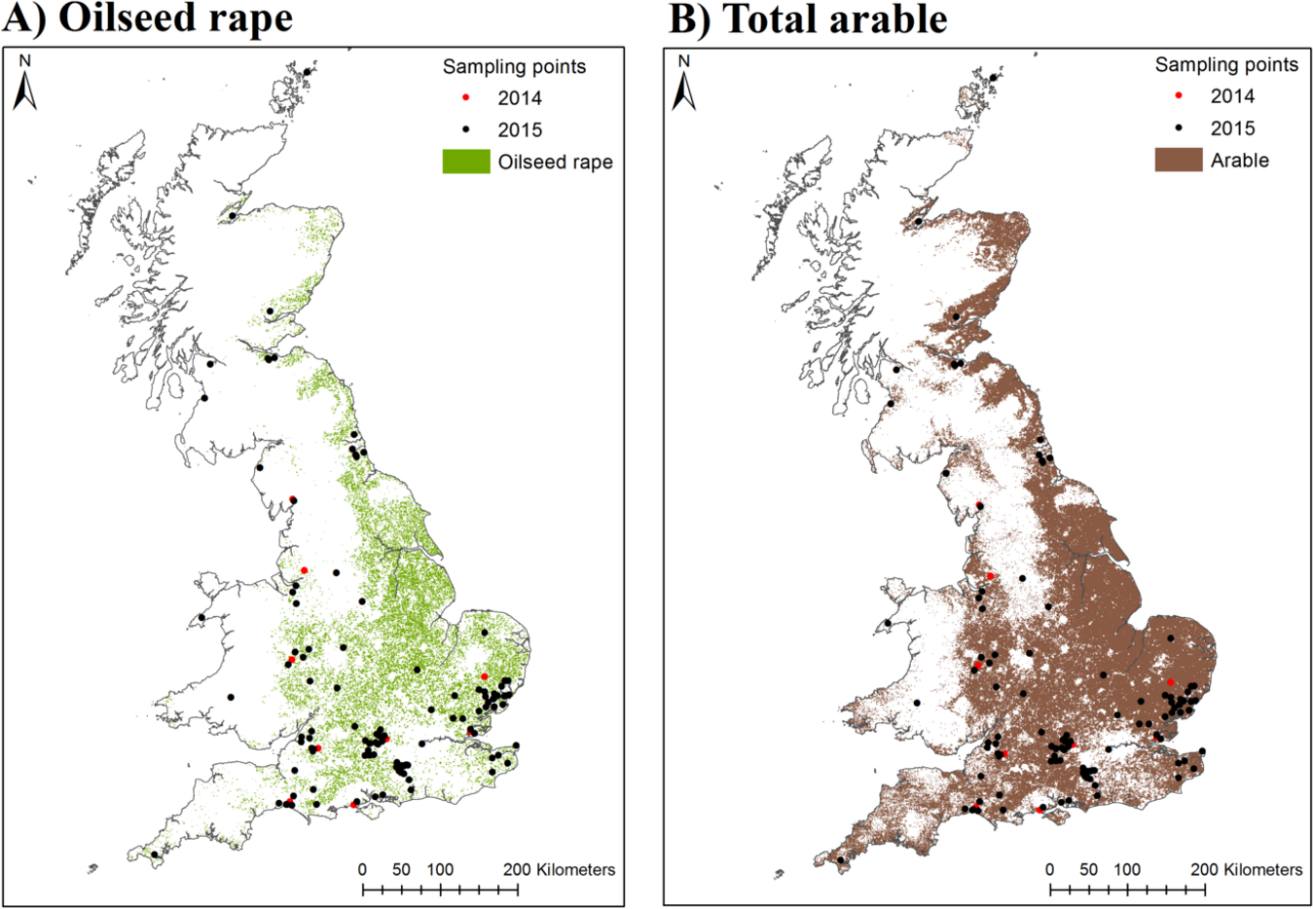
Location of UK honey samples. The two maps show the location of honey samples collected in 2014–15 superimposed over the cover of all arable crops (A) and oilseed rape (B).

Each sample comprised blended honey originated from a harvest from a single location of the supers (frames) of individual hives or hives (typically 2–3). Insufficient records are kept to distinguish between honey samples originating from single or multiple hives. However within a single site hives would be within 5–10 m of each other. Where honey was blended from hives in several locations this was excluded from the analysis. Samples from 2012–2013 were excluded due both to their age and limited replication. Further samples were removed from the data set where exact information could not be provided as to the location of the original hive (for example where honey was amalgamated from hives in multiple locations). A small number of samples from Ireland were also excluded as they were outside of the geographical range for spatial information on crop coverage. This resulted in a sub-set of 130 samples from individual beekeepers (N_2014_ = 21, N_2015_ = 109). There was a good spatial distribution of samples, with honey collected from over 20 counties (Fig 1).

The modal month of honey collection was August, although samples were collected from May to September (Table B in S1 Appendix). It should be noted that while many beekeepers regularly remove honey from hives through the season (to increases the total yield) this is not a universal practice. It is possible that honey samples collected from periods after oilseed rape flowering ended may have included honey originating from while it was in flower [25]. For this reason the harvest month reported for individual honey samples should be considered only as an indication of the time of year when it was sourced.

### Residue analysis

Samples of honey were analysed to quantify concentrations of clothianidin, thiamethoxam (UKAS accredited ISO17025:2005 standards) and imidacloprid. In all cases honey had been collected using normal bee keeping practice, whereby honey was spun from hive supers (frames containing honey combs). This collected honey that was homogenized and stored for subsequent human consumption. From this honey a 1.5 ml sub-sample was collected and stored at -80°C, although prior to this it was kept under conditions typical for food storage and so not frozen.

Determination of the wet weight concentration of the three neonicotinoid compounds was undertaken using liquid chromatography coupled to a triple quadrupole ‘Quantum Ultra TSQ’ mass spectrometer (Thermo Fisher Scientific, Hemel Hemsptead; UK). This was interfaced using an ion max electrospray ionisation (ESI). Analyte separation was performed on a Phenomenex Synergi Fusion column (2.5 μm particle size, 50 mm x 2mm I.D., Phenomenex) using a H2O:MeOH mobile phase gradient. In each case residues were compared to spiked samples (0.1g) labelled with internal neonicotinoid standards (Thiamethoxam, Clothianidin, Imidacloprid). Quantification of neonicotinoid residues were based on their respective response factor to these internal standards. Chromatographic peaks were integrated using the ICIS algorithm, which was also used to generate linear calibration curves based on a 1/X weighting (*R*^*2*^>0.99). The performance of the method was assessed in terms of the limit of detection (LoD = 0.38 ng g^-1^) and recovery of the internal standards for the analytes. The LoD was derived as three times the signal to noise ratio including an assessment of the expanded uncertainty of the method [26]. The LoQ (limit of quantification) was calculated as the LoD plus the calculated expanded uncertainty of the method (LoQ = 0.53 ng g^-1^). Honey residue that contained neonicotinoids above the limit of quantification were given a value equal to their detected concentration. When honey samples contained residues below the limit of quantification, but greater than the limit of detection they were allocated a fixed values equivalent to the limit of detection (LoD = 0.38 ng g^-1^). We used this approach as within this range neonicotinoid residues are detectable (i.e. above the LoD), but are at concentration too low to provide an exact residue value (i.e. below the LoQ). This approach follows the same principals as that used in comparable studies looking at pesticide residues in honeybee colonies [19, 20, 22]. Residues below the limit of detection were considered to have a zero neonicotinoid residues, although it is possible that some very low levels of neonicotinoids were present in the range of 0.0–0.38 ng g^-1^. It should be noted that neonicotinoid concentrations in honey would not be the same as that found in the nectar (e.g. from oilseed rape or wild flowers) which the bees collected and used to create it. When stored as honey the water content of nectar is reduced as part of the preservation process. In addition, the mixing of contaminated and uncontaminated nectar by bees may lead to the dilution of neonicotinoid residues in stored honey [27].

### Agricultural land use as a predictor of neonicotinoid exposure risk

Oilseed rape is the principal mass flowering crop grown in the UK to which neonicotinoid seed treatments have been applied and as such represents the most likely mechanisms through which honeybees would be exposed to these pesticides before the EU moratorium [4, 6–8, 28, 29]. We focus on this crop as in addition to is dominant land cover it also provides a functionally homogenous mechanism of exposure in terms of flower type and broad levels of attraction to honeybees [30]. In 2014 (the final pre-moratorium year) a total of 674,580 hectares of oilseed rape (98% winter sown in 2013) were harvested in the UK. Of this 95.0% received some form of seed treatment (including non-neonicotinoid products), with thiamethoxam (50.9% of seed treated crop) and clothianidin (33.3% of seed treated crop) being the most frequently used [31]. The use of imidacloprid in the UK had largely stopped by 2014, so that only 0.3% of seed treated oilseed rape was treated with imidacloprid in this year [31]. With the exception of small areas of experimentally treated crops [9], neonicotinoids were not applied as seed treatments in 2015 to any mass flowering crop in the UK (Table A in S1 Appendix). None of these areas were within 10 km of any of the collected honey samples. It should be noted that other mass flowering crops are sown in the UK, although all are much less frequently grown than oilseed rape. For example field beans (*Vicia faba*) grown on only 3.0% of the cropped area [29]. Also, neonicotinoid seed treatments were applied only to oilseed rape and not field beans [27, 32].

While there is a reasonable expectation that residues of neonicotinoids in honey would be predicted by the cover of oilseed rape in years prior to the EU moratorium (Table B in S1 Appendix, Fig A in S1 Appendix), the extent to which soil contamination resulted in post-moratorium contamination of honey remains unclear. If neonicotinoids persist in soil there is considerable potential that they may be found in untreated mass flowering crops growing on land where seed treated crops were grown in previous years (including winter sown cereals). To assess potential routes of exposure linked to arable land use, we correlated the summed residues of all three neonicotinoid products (clothianidin, thiamethoxam and imidacloprid) in honey to the cover of oilseed rape, winter sown cereals and total arable crops. This was done for honey samples from 2015 when the effective EU moratorium on neonicotinoid use in mass flowering crops was in place.

To derive information on the cover of crops we used the 2015 CEH Land Cover Plus: Crops map (NERC CEH) [33] in ESRI ArcGIS v10.4 (ESRI, Redlands, CA). This is the first high resolution map of UK cropping, classifying *c*. two million land parcels based on the existing UK Land Cover Map (LCM) (Fig 1). The minimum mapping unit for the crop map is represented by 2 ha land parcels and is derived from a combination of Copernicus Sentinel-1 C-band SAR (Synthetic Aperture Radar) and Sentinel-2 optical data. Using this map we assessed the cover of oilseed rape, winter sown cereals and total arable crops for a 2 km radius surrounding the location of hives from which a honey sample was provided. This radius reflects commonly reported mean foraging ranges of honeybees, although they are capable of foraging over larger distances [34, 35].

### Statistical analysis

Honey samples where neonicotinoids residues did not exceeded the limit of detection (LoD = 0.38 ng g^-1^) were considered to have zero neonicotinoid residues. The resulting residue data was dominated by these zeroes (72.3% of values were non-detections), although was also non-negative and continuous. The presence of zero-inflation was potentially the result of two unquantified factors: 1) a lack of neonicotinoid residues as they were not present in the landscape or hive; or 2) a failure to detect residues that were present in the hive, but not the particular sub-sample of analysed honey, or those that were present but had degraded below the limit of detection. Modelling such ‘zero-inflation’ is commonplace for discrete data, but has only recently begun to be performed for continuous distributions [36, 37]. We analysed the zero-inflated data using a Tweedie distribution [38] modeled within the package FishMod implemented in R version 3.1.2 [32, 36, 39]. The method used a log link function to keep predicted values above zero and extends the Tweedie distribution so that the expected value of the *i*^th^ observation of the response (μ_i_) predicted by the covariate (*y*_i_) is given by the below equations.

$$Log(µ)=a^{T}X$$

$$Var(y_{i})=\varphi µip$$

Where parameter *a* relates the neonicotinoid residue values to a vector *X* of appropriate covariates. The additional parameters *φ* and *p* set the shape of the curve for 1<*p*<2 and result in a Poisson mixture of gamma distributions that serves to produce the ‘spike’ at zero [36].

Within this framework we tested for the correlated response of the combined residues of all three neonicotinoid products detected in honey (NNI = clothianidin + thiamethoxam + imidacloprid) to agricultural land uses. Three separate models considered the cover of oilseed rape (a key foraging crop grown on soils likely to contain neonicotinoid residues), cover of winter sown cereals (to which neonicotinoid seed treatments could have been applied in 2015) or total arable cover (total land on which neonicotinoid seed treatments may have been used over previous years). Strong correlations between the three agricultural land uses prevented their inclusion in an overall additive model (Table C in S1 Appendix). In addition to the responses to landscape, we also used the same approach to assess if combined neonicotinoid residues changed in response to the month from which the honey was harvest in 2015. In each case we use likelihood ratio tests with a *χ*^*2*^ test statistic to assess if the inclusion of the landscape structure explained variation in neonicotinoid residues by comparing μ = a_0_ + *a*_1_*X to an intercept only model of μ = a_0_, where X = land use cover. To test for underlying spatial structure in the data we performed a Moran’s I test of the raw neonicotinoid residue data. Goodness of fit was also assessed by examination of model residuals. Due to the limited availability of samples from the pre-moratorium year (2014: N = 21) we focus the analysis in the main paper on data from the 2015 (N = 111) field season. This represents the first year of the neonicotinoid moratorium. However, for the 2014 analysis based on this limited data set see Fig A and Table C in S1 Appendix. By separating the analysis into separate years we account for potential confounding differences in the breakdown of neonicotinoid residues in the honey between 2014 and 2015.

## Results

### Effectiveness of the moratorium in reducing neonicotinoid residues in honey

**Fig 2:**
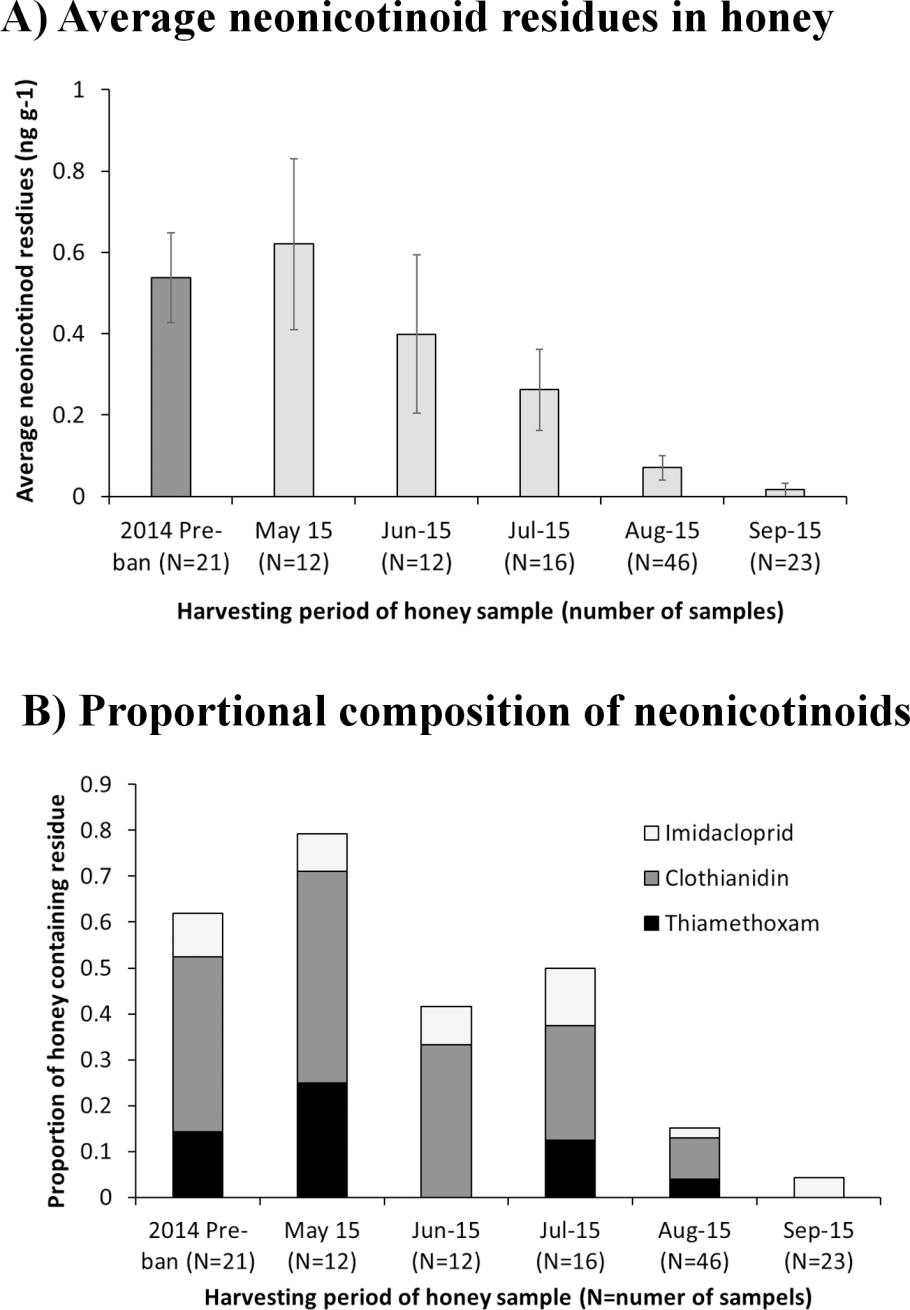
Change in neonicotinoid residues in honey pre- and post-moratorium. The first graph (**A**) shows the change in average (±SE) combined (clothianidin, thiamethoxam and imidacloprid) residues found in honey over time. Due to the limited number of samples the pre-moratorium period is combined into a single value. The second graph (**B**) shows how the proportion of honey samples containing neonicotinoid residues changed over time. Note that residues of more than one neonicotinoid type may appear in a single honey sample. As such the proportion of samples containing either clothianidin, thiamethoxam or imidacloprid has been scaled so that when combined it does not exceed the proportion of honey samples containing neonicotinoid residues of any type. Where N = the number of honey samples for a particular time period.

Overall 130 honey samples were analysed (N_2014_ = 21; N_2015_ = 109). The small number of samples from 2014 reflect the availability of honey stocks from older years relative to honey harvested in 2015 (Tables 1 & 2). A greater proportion of honey from 2014 had been sold for consumption and so was not available for analysis. Following the moratorium, average concentrations of all neonicotinoid in contaminated honey samples declined through 2015 (*χ*^*2*^_*4*_ = 31.9, p<0.001, Fig 2A), although combined median residues were only above zero in May of that year (Table 1). Overall the concentrations of clothianidin, thiamethoxam and imidacloprid residues within honey were typically low and did not exceed 1.69 ng g^-1^ for any given product. The combined residues of all three products did not exceeding 1.99 ng g^-1^ in a honey sample in 2015. However, across the three products there was little difference in the maximum residue concentration in the post moratorium period, with the values ranging from 1.41–1.69 ng g^-1^ (Table 1). The likelihood of honey containing neonicotinoid residues was higher before the moratorium than after it, with 52.3% of samples from 2014 containing residues of either clothianidin, thiamethoxam or imidacloprid, compared to the 22.9% in 2015 (Tables 1 & 2; Fig 2). However, following the implementation of the moratorium in 2015 residues were most frequently detected in honey collected from the period of oilseed rape flowering (May to July) rather than the months following this (Fig 2B). Occasionally, honey samples contained two of the three neonicotinoid compounds (3.8%), although no sample contained all three (Table B in S1 Appendix). The most frequently identified neonicotinoid was clothianidin (Table 1), which was in 72.0% of samples testing positive for neonicotinoids in 2014 (pre-moratorium) and 38.1% of samples in 2015 (post-moratorium). Thiamethoxam and imidacloprid were less common, occurring in 14–28% of neonicotinoid-contaminated honey samples in either year.

**Table 1:** Summary of neonicotinoids residues found in honey pre- and post the EU moratorium.

Date	Number of honey samples	Average combined NNI residue (ng g^-1^ w/w)	Maximum combined NNI residue (ng g^-1^ w/w)	Median combined NNI residue (ng g^-1^ w/w)	Proportion of honey containing neonicotinoids residues above LOD (0.38 ng g^-1^ w/w)			
					*NNI*	*TMX*	*CTD*	*IMI*
**2014 (all)**	21	0.43 (SE = 0.12)	2.00	0.38	0.52	0.14	0.38	0.10
**2015 (all)**	109	0.19 (SE = 0.04)	1.99	0.00	0.23	0.06	0.17	0.06
**May-15**	12	0.62 (SE = 0.21)	1.79	0.62	0.66	0.25	0.50	0.08
**Jun-15**	12	0.40 (SE = 0.19)	1.99	0.00	0.33	0.00	0.33	0.08
**Jul-15**	16	0.26 (SE = 0.10)	1.10	0.00	0.38	0.13	0.25	0.13
**Aug-15**	46	0.07 (SE = 0.03)	0.76	0.00	0.13	0.04	0.09	0.02
**Sep-15**	23	0.02 (SE = 0.02)	0.38	0.00	0.04	0.00	0.00	0.04

Summary statistics for the combined (NNI) residues of thiamethoxam (TMX), clothianidin (CTD and imidacloprid found within honey pre- (2014) and post (2015) the implementation of the EU moratorium on their use in mass flowering crops. Seasonal changes in residues post moratorium are also shown. Note that residues of more than one neonicotinoid compound may be found in the same sample of honey.

**Table 2:** 

	Thiamethoxam	Clothianidin	Imidacloprid
**2014**	Ave. = 0.1 ng g^-1^ (SE = 0.13) Max = 1.41 ng g^-1^	Ave. = 0.28 ng g^-1^ (SE = 0.14) Max = 1.02 ng g^-1^	Ave. = 0.05 ng g^-1^ (SE = 0.09) Max = 0.64 ng g^-1^
**2015**	Ave. = 0.04 ng g^-1^ (SE = 0.02) Max = 1.41 ng g^-1^	Ave. = 0.11 ng g^-1^ (SE = 0.03) Max = 1.69 ng g^-1^	Ave. = 0.03 ng g^-1^ (SE = 0.02) Max = 1.61 ng g^-1^
**May-15**	Ave. = 0.24 ng g^-1^ (SE = 0.14) Max = 1.41 ng g^-1^	Ave. = 0.45 ng g^-1^ (SE = 0.17) Max = 1.69 ng g^-1^	Ave. = 0.03 ng g^-1^ (SE = 0.03) Max = 0.38 ng g^-1^
**Jun-15**	Ave. = 0.00 ng g^-1^ (SE = 0) Max = 0.00 ng g^-1^	Ave. = 0.27 ng g^-1^ (SE = 0.2) Max = 1.37 ng g^-1^	Ave. = 0.13 ng g^-1^ (SE = 0.21) Max = 1.61 ng g^-1^
**Jul-15**	Ave. = 0.05 ng g^-1^ (SE = 0.09) Max = 0.38 ng g^-1^	Ave. = 0.16 ng g^-1^ (SE = 0.14) Max = 0.72 ng g^-1^	Ave. = 0.06 ng g^-1^ (SE = 0.1) Max = 0.53 ng g^-1^
**Aug-15**	Ave. = 0.02 ng g^-1^ (SE = 0.02) Max = 0.69 ng g^-1^	Ave. = 0.04 ng g^-1^ (SE = 0.02) Max = 0.58 ng g^-1^	Ave. = 0.01 ng g^-1^ (SE = 0.01) Max = 0.38 ng g^-1^
**Sep-15**	Ave. = 0.00 ng g-1 (SE = 0) Max = 0.00 ng g-1	Ave. = 0.00 ng g^-1^ (SE = 0.00) Max = 0 ng g^-1^	Ave. = 0.02 ng g^-1^ (SE = 0.06) Max = 0.38 ng g^-1^

Summary of neonicotinoids residues found in honey. Mean and maximum recorded concentrations for individual compounds (thiamethoxam, clothianidin and imidacloprid) within honey from 2014–2015 are provided. Due to the zero inflate nature of the data median residue values for individual compounds were always zero.

### Oilseed rape crops as a predictor of neonicotinoid residues in honey following the moratorium

**Fig 3:**
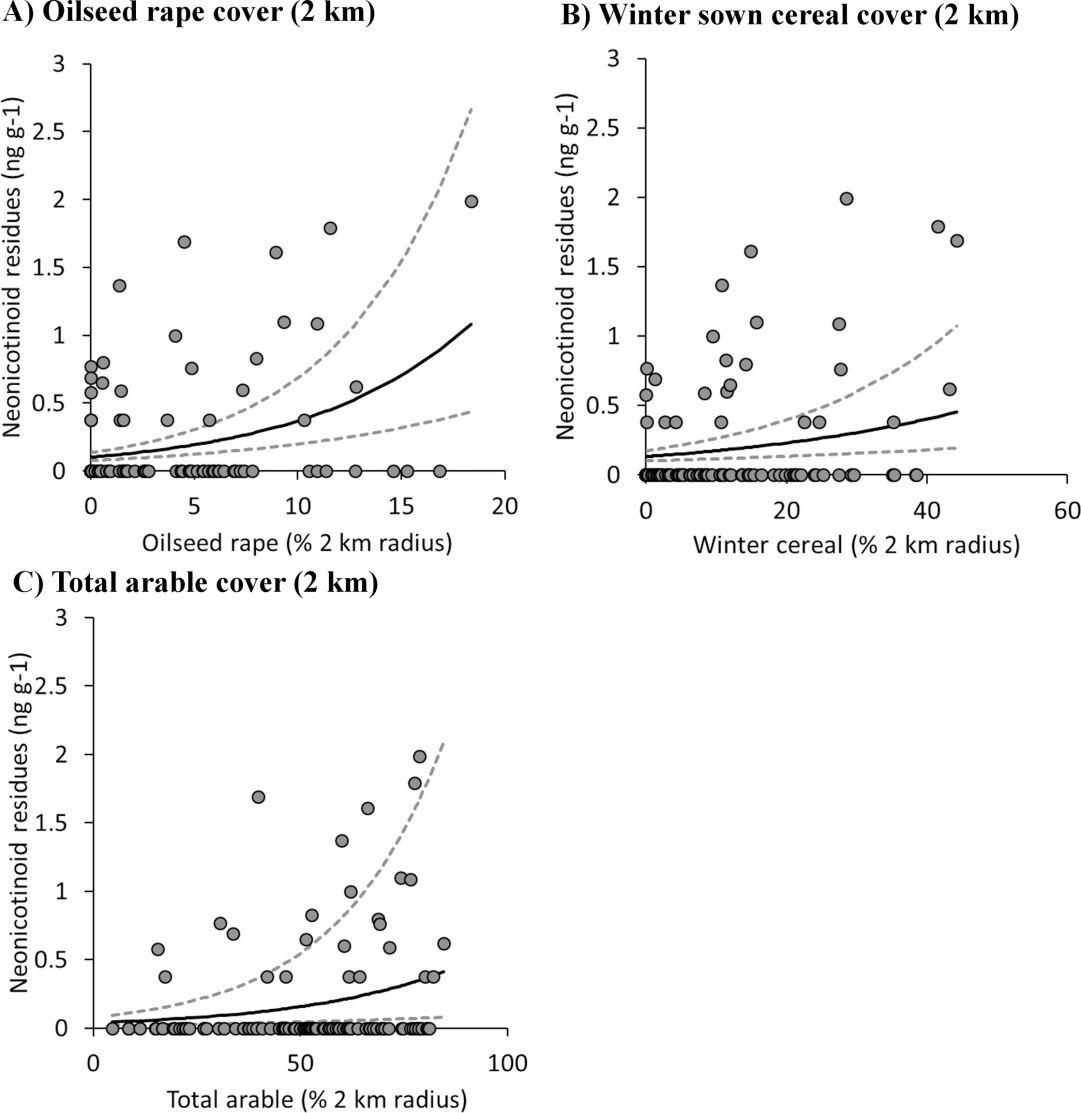
Response of combined neonicotinoid residues in honey to land use. The graphs show the back transformed model predictions (±SE) for the response of combined neonicotinoid residues found in honey to (**A**) oilseed rape cover, (**B**) winter sown cereals and (**C**) total arable cover. All honey was collected in 2015 during the first year where the use of neonicotinoids seed treatments had been banned on mass flowering crop in the EU. All percentage covers are within 2 km radii of individual hives. Neonicotinoid residues represent the combined concentration of imidacloprid, thiamethoxam and clothianidin.

Although neonicotinoid residues declined from May to September in 2015, there were positive correlations between three types of agricultural land use and the combined neonicotinoid (clothianidin, thiamethoxam and imidacloprid) concentrations in all honey samples from that year (Table 3). Positive correlations were with the cover of oilseed rape (Fig 3A), the cover of winter sown cereals (Fig 3B) and the combined cover of all arable crops (Fig 3C) (Table 3). The cover values of the different land use types were strongly correlated and so could not be included in a single additive model (Table B in S1 Appendix). However, comparing models using single land use types showed that the model using cover of oilseed rape as a predictor of neonicotinoid residues in honey had the lowest *AICc* values and thus the greatest explanatory power (Table 3). There was no evidence of spatial autocorrelation in the concentrations of neonicotinoids in honey from either the pre-moratorium (Moran’s I test: observed / expected I = -0.13/-0.05, p = 0.42) or post-moratorium period (Moran’s I: 0.019/-0.009, p = 0.34). A correlation between oilseed rape cover and the residues of neonicotinoids in honey from the pre-moratorium period was also found for the limited number of 2014 samples taken before the moratorium (Fig A and Table D in S1 Appendix).

**Table 3:** Likelihood ratio tests for honey residue responses to oilseed rape crop cover.

Model	Log Lik.	Para.	φ	*p*	n	*AICc*	LRT
μ = a_0_ + *a*_1_*OSR	-64.87	4	0.92	1.12	109	139.6	χ^2^_1_ = 12.4, p<0.001
μ = a_0_ + *a*_1_*Winter cereals	-69.02	4	1.03	1.14	109	146.4	χ^2^_1_ = 4.08, p = 0.04
μ = a_0_ + *a*_1_*Arable	-67.73	4	1.01	1.13	109	143.8	χ^2^_1_ = 6.66, p = 0.01

The significance of the response of combined neonicotinoid residues detected in honey (NNI = clothianidin + thiamethoxam + imidacloprid) in the first year (2015) of the European Union moratorium on the use of neonicotinoids. Combined neonicotinoids residues are correlated with potential agricultural sources of these pesticides in the form of oilseed rape cover (OSR), winter sown cereal cover and total arable cover. Likelihood Ratio Tests assess whether these responses explain more variance than intercept only model. Log Likelihood, number of parameters (including φ and *p* for the Tweedie distribution), sample size (n) and *AICc* are also provided.

## Discussion

This national survey detected residues of neonicotinoid in over one fifth of samples originating from honey harvested in the first year of an EU moratorium on the use of these pesticides on mass flowering crops. While this represented a reduction in the frequency of honey contaminated with neonicotinoids compared to the pre-moratorium period (as identified both here and from pre-moratorium surveys in France [20]), this suggests that these pesticides remain prevalent in the farming environment. Indeed the persistence of residues was highlighted by the detection of imidacloprid in honey at a rate disproportional to its use in 2014; by which time it had largely been replaced in the UK by clothianidin and thiamethoxam [31]. The combined concentrations of neonicotinoids residues (imidacloprid, clothianidin and thiamethoxam) never exceeding 2.0 ng g^-1^ in samples from either the pre- or post-moratorium period. This represents comparable concentrations of neonicotinoids to those reported from worldwide honey surveys of neonicotinoids, although mean concentrations found here were lower than their reported 1.8 ng g^-1^ [24]. Importantly, these concentrations pose no risk to human health being more than two orders of magnitude below the 500 ng g^-1^ maximum residue level permitted in honey intended for human consumption [40].

Even for honeybees that consume stored honey these concentrations are below those that cause acute mortality. For example, honeybees exposed to our highest recorded residues would receive 0.08 ng day^-1^ bee^-1^ based on the daily consumption of 40 mg of honey [41]. This falls well below acute oral LD_50_ values [12]. Under controlled regulatory conditions quantification of long term (> 10 day) chronic effects of neonicotinoids (e.g. *LC*_*50*_ values) are hard to achieve due to methodological issues relating to keeping control populations of bees alive. For this reason, the implications of low level residues in honey are not easy to assess in terms of their impacts under field conditions. However, these neonicotinoid concentration can also be considered in terms of a hazard quotient (HQ), which provide an indication of the potential risk from neonicotinoids consumed in honey relative to acute oral toxicity values [12, 21–23]. When this is derived we detect a peak value of 480, which while an order of magnitude lower than values for imidacloprid reported by Tosi, Costa (22) (HQ = 5054) and Stoner and Eitzer (21) (HQ = 17,949), is still equivalent to a bee consuming 12% of its LD_50_ a day. This suggests a potential risk of long-term low-level exposure over the course of an entire flowering season [4, 5]. Importantly, residues in honey and pollen, equivalent to the concentrations identified here, have been shown to reduce colony viability in overwintering honeybees [9]. Long term sub-lethal effects of neonicotinoids have also been reported in response to concentrations equivalent to those we found in UK honey [24]. While the impacts of the residue in honey identified here should be considered with caution, an absence of information on the consequences of long-term chronic effects of neonicotinoids remains a significant evidence gap for understanding their implications.

An important finding of the study was that concentrations of neonicotinoids in honey did decline from May to September following the implementation of the moratorium. This could be due to either the gradual loss of persisting neonicotinoid residues in the soil, or due to the short early season flowering of oilseed rape providing an important mechanisms of exposure to honeybees of neonicotinoid soil residues. Ultimately any link to land use presented here is correlative and as such can only be used to infer possible mechanisms thorough which foraging honeybee may become exposed to neonicotinoid residues following the moratorium. However, clarification of the mechanisms is required if better stewardship of these pesticides is to be achieved. We hypothesized two non-exclusive exposure routes in agricultural systems. These are: 1) the presence of neonicotinoids in mass-flowering crops grown in soils contaminated with these compounds, and 2) the presence of neonicotinoids in wildflowers grown on neonicotinoid contaminated soils though previous use or the drift of contaminated dust or surface water from treated crops [5, 14–16, 18]. The latter of these hypotheses can also be considered in terms of the underlying mechanism of soil contamination, which may be driven to a far greater extent by accidental contamination of non-crop areas though dust when sowing or movement of surface water from areas of treated crop [5, 14–16, 18]. Note, that the importance of drift is considered to be very low for winter sown cereals, although contamination risk from surface water remains a potential issue [10, 18]. Previous work has also suggested that due to the relatively short flowering period of oilseed rape, wild plants found throughout the growing season may pose a risk to bees if non-target neonicotinoids are present in their pollen and nectar [15]. However, the current analysis focuses on dominant crops likely to be acting as a primary source of exposure in terms of total land cover and historical and ongoing practices of neonicotinoid use. Agricultural systems are complex interacting environments and simple metrics of land cover as used here can only provide indications of potential routes of exposure for these pesticides.

While our results do not preclude the potential for wild flowers to provide a risk of exposure to neonicotinoid for bees, we would argue that our evidence suggests that the identification of non-target neonicotinoids in mass-flowering crops may pose the greater risk. This reflects the relatively large areas over which these crops are sown and the continued use of neonicotinoid seed treatments on cereals that may precede them in a cropping rotation [29]. Further, the cover of untreated oilseed rape was the better predictor of the relationship between agricultural land use and neonicotinoid residues in honey when compared to the cover of either total arable (> 5 *AICc* difference) or winter sown cereals (>8 *AICc* difference). The importance of oilseed rape as mechanism of continued exposure risk for honeybees is also supported by the number of samples containing residues predominantly associated with the peak period of oilseed rape flowering (May-June). Early season exposure to neonicotinoids, which would occur where neonicotinoids were found in untreated oilseed rape, may pose a particular risk to honeybees as this coincides with the period where colonies are small and queens show their highest levels of vulnerability [42]. The predominance of early season neonicotinoid residues interestingly contrasts with an Italian study that found that peak concentrations of all pesticide residues are associated with the summer months [22]. Although we propose the early season prevalence of residues as a link to oilseed rape flowering, it should be noted that there is some evidence that autumn supplementary feeding with sugar syrup (a practice used to promote greater honey yields the following summer) may cause bees to move syrup stored from brood chamber into the supers over the winter when they need that space for rearing brood. We therefore cannot exclude the possibility that some of the honey collected in the spring of 2015 may contain honey from the previous year where it was stored in the brood chamber over the winter period [25].

## Conclusions

The sustainable use of pesticides remains dependent on robust risk assessments that protect not just human health, but also the environment and associated ecosystem processes [43, 44]. The results of this national survey suggests that the EU moratorium on neonicotinoid use for mass-flowering crops have been only partially effective in reducing exposure risk to bees. There was evidence for continued neonicotinoid residues in honey following the moratorium, although the occurrence of these residues have declined in the months following the implementation of the ban. This risk of exposure is likely linked to persistent soil residues in the extensive areas where oilseed rape is grown. It is important to emphasize that the relationships presented in this study are correlative. While the presence of residues after the ban and our identification of a correlation between oilseed rape cover add weight to soil residues as a mechanism of exposure risk for honeybees to neonicotinoids they cannot confirm it directly. Research is ultimately required to quantify how long neonicotinoid residues persist under field conditions, in particular by providing quantification of those conditions or soil types where degradation rates are reduced. Where the persistence of soil residues following neonicotinoids use is a problem then restricting application of seed-treatments in crops that precede a mass flowering species, like oilseed rape, may help to reduce the presence of these pesticides in pollen and nectar. However, it remains to be seen if residues in honey continue to persist over time under current permitted neonicotinoid use and agricultural rotations in the EU.

## Supporting information

S1 Appendix Supporting figures and tables. Figures and tables describing raw data and 2014 pre-moratorium results for neonicotinoid residues in honey.
(DOCX)
