# Supplementary material for: Neonicotinoid residues in UK honey despite European Union moratorium
Source: PLoS One. 2018 Jan 3;13(1):e0189681. doi: 10.1371/journal.pone.0189681 (PMC5751988; doi:10.1371/journal.pone.0189681)
Supplement: S1 Appendix — Figures and tables describing raw data and 2014 pre-moratorium results for neonicotinoid residues in honey. (DOCX) [file pone.0189681.s001.docx]

S1 Appendix: Other figures and tables. Figures and tables describing raw data and 2014 pre-moratorium results for neonicotinoid residues in honey.

**Fig A. Effect of arable crop cover on neonicotinoid residues in honey**. This is based on the overall 2014 data set for honey and shows the response of combined neonicotinoid residues (clothianidin, thiamethoxam and imidacloprid) to the cover of oilseed rape within 2km of honeybee hives. There was no evidence of spatial autocorrelation in the combined residues of neonicotinoid across the overall data set (Moran’s I test: observed / expected I = -0.13/-0.05, p=0.42)


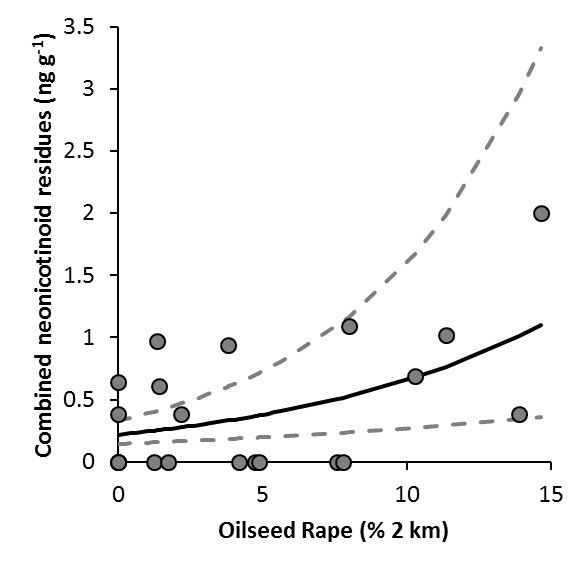


**Table A.** **EU Neonicotinoid moratorium time line.**  This table provides a time line of when the EU moratorium was initiated and the impacts that this had on neonicotinoid use on oilseed rape in GB. This is based on the 2017 House of Commons Briefing paper number 06656 [10].

| **Year** | **Season** | **Event** |
| --- | --- | --- |
| 2013 | Autumn | Winter oilseed rape treated with neonicotinoid seed treatments sown for harvest in 2014. |
| 2013 | December | EU moratorium on neonicotinoid use in mass flowering crops instigated. Winter cereals are not part of the moratorium restrictions. |
| 2014 | Spring/summer | Oilseed rape treated with neonicotinoids in 2014 both flowers and is harvested. |
| 2014 | Autumn | Winter oilseed rape receiving no neonicotinoid seed treatment (due to moratorium) is sown for harvest in 2015. |
| 2015 | Spring/summer | Untreated oilseed rape flowers and is harvested. |
| 2015 | July | Emergency derogations are granted for some areas of UK allowing the use of neonicotinoids seed treatments on oilseed rape. This equated to approximately 5% of the sown area of crop as was restricted to the countries of Cambridgeshire, Suffolk, Hertfordshire and Bedfordshire. |
| 2015 | Autumn | Winter oilseed rape treated receiving no neonicotinoid seed treatment due to moratorium is sown for harvest in 2015. However, due to exemptions granted neonicotinoid treated oilseed rape is four counties in the UK. |
| 2016 | Spring/summer | Untreated oilseed rape flowers and is harvested with the exception of those areas treated with neonicotinoids as part of the exemptions granted in 2015. |

**Table B.** **Raw data providing for neonicotinoid residues in honey.** Both combined (NNI) and separate thiamethoxam, (TMX), clothianidin (CTD) and imidacloprid (IMI) neonicotinoid residues are given. The spatial location of each beekeeper is anonymized to a 10 km grid square resolution. The calendar month of honey harvest. Residues below the limit of quantification (LOQ=0.53 ng g^-1^ w/w) but above the limit of detection (LOD=0.38 ng g^-1^ w/w) are set to the LOD, values below the LOD are set to 0.00.

| **Year** | **Month** | **Easting** | **Northing** | **Arable cover (% in 2 km radius)** | **Oilseed rape cover (% in 2 km radius)** | **Winter cereals cover (% in 2 km radius)** | **TMX (w/w ng g^-1^)** | **CTD (w/w ng g^-1^)** | **IMI (w/w ng g^-1^)** | **NNI (w/w ng g^-1^)** |
| --- | --- | --- | --- | --- | --- | --- | --- | --- | --- | --- |
| 2014 | May | 441900 | 177100 | 80.12 | 10.28 | 24.60 | 0.00 | 0.69 | 0.00 | 0.69 |
| 2014 | May | 596500 | 259600 | 83.47 | 14.67 | 22.89 | 1.41 | 0.59 | 0.00 | 2.00 |
| 2014 | May | 439800 | 549800 | 57.19 | 7.78 | 14.52 | 0.00 | 0.00 | 0.00 | 0.00 |
| 2014 | May | 427100 | 94300 | 50.93 | 3.81 | 13.44 | 0.00 | 0.94 | 0.00 | 0.94 |
| 2014 | June | 348300 | 489100 | 51.72 | 0.00 | 1.55 | 0.00 | 0.00 | 0.00 | 0.00 |
| 2014 | June | 577900 | 188800 | 25.78 | 1.23 | 1.07 | 0.00 | 0.00 | 0.00 | 0.00 |
| 2014 | June | 447700 | 173800 | 66.86 | 11.37 | 18.14 | 0.00 | 1.02 | 0.00 | 1.02 |
| 2014 | June | 346900 | 281800 | 88.11 | 4.19 | 34.57 | 0.00 | 0.00 | 0.00 | 0.00 |
| 2014 | June | 363300 | 397000 | 52.17 | 2.18 | 14.42 | 0.00 | 0.38 | 0.00 | 0.38 |
| 2014 | July | 347800 | 368900 | 71.52 | 1.42 | 8.32 | 0.00 | 0.61 | 0.00 | 0.61 |
| 2014 | July | 469400 | 179300 | 56.08 | 4.75 | 16.10 | 0.00 | 0.00 | 0.00 | 0.00 |
| 2014 | Aug. | 381000 | 167200 | 60.48 | 7.60 | 12.22 | 0.00 | 0.00 | 0.00 | 0.00 |
| 2014 | Aug. | 627900 | 236100 | 52.70 | 7.99 | 11.30 | 0.00 | 0.71 | 0.38 | 1.09 |
| 2014 | Aug. | 622300 | 254700 | 79.51 | 13.91 | 33.50 | 0.38 | 0.00 | 0.00 | 0.38 |
| 2014 | Aug. | 491600 | 134600 | 22.19 | 0.00 | 3.22 | 0.00 | 0.00 | 0.00 | 0.00 |
| 2014 | Aug. | 429800 | 546700 | 59.07 | 4.87 | 10.50 | 0.00 | 0.00 | 0.00 | 0.00 |
| 2014 | Aug. | 437800 | 357200 | 50.67 | 0.00 | 3.50 | 0.38 | 0.00 | 0.00 | 0.38 |
| 2014 | Aug. | 583400 | 186600 | 16.71 | 0.00 | 0.05 | 0.00 | 0.00 | 0.64 | 0.64 |
| 2014 | Aug. | 623100 | 235100 | 66.67 | 1.74 | 16.43 | 0.00 | 0.00 | 0.00 | 0.00 |
| 2014 | Sept. | 344400 | 98400 | 73.14 | 0.00 | 8.08 | 0.00 | 0.00 | 0.00 | 0.00 |
| 2014 | Sept. | 425600 | 553600 | 51.72 | 1.36 | 21.44 | 0.00 | 0.97 | 0.00 | 0.97 |
| 2015 | May | 555700 | 205700 | 39.69 | 4.48 | 44.25 | 0.00 | 1.69 | 0.00 | 1.69 |
| 2015 | May | 497200 | 145500 | 79.36 | 14.61 | 0.00 | 0.00 | 0.00 | 0.00 | 0.00 |
| 2015 | May | 457000 | 185400 | 84.49 | 12.82 | 43.18 | 0.00 | 0.62 | 0.00 | 0.62 |
| 2015 | May | 368900 | 295300 | 81.99 | 5.71 | 35.23 | 0.00 | 0.38 | 0.00 | 0.38 |
| 2015 | May | 626700 | 254800 | 77.51 | 11.56 | 41.49 | 1.41 | 0.00 | 0.38 | 1.79 |
| 2015 | May | 608900 | 220900 | 68.71 | 0.60 | 14.17 | 0.00 | 0.80 | 0.00 | 0.80 |
| 2015 | May | 501200 | 113400 | 62.03 | 4.08 | 9.46 | 0.00 | 1.00 | 0.00 | 1.00 |
| 2015 | May | 439800 | 549800 | 57.19 | 7.78 | 14.52 | 0.00 | 0.00 | 0.00 | 0.00 |
| 2015 | May | 431000 | 98100 | 30.50 | 0.00 | 0.02 | 0.77 | 0.00 | 0.00 | 0.77 |
| 2015 | May | 596300 | 240300 | 78.61 | 4.35 | 24.19 | 0.00 | 0.00 | 0.00 | 0.00 |
| 2015 | May | 588900 | 242800 | 66.16 | 8.93 | 14.87 | 0.70 | 0.91 | 0.00 | 1.61 |
| 2015 | May | 607600 | 231200 | 61.06 | 0.36 | 5.06 | 0.00 | 0.00 | 0.00 | 0.00 |
| 2015 | June | 316400 | 670700 | 61.50 | 5.38 | 20.92 | 0.00 | 0.00 | 0.00 | 0.00 |
| 2015 | June | 459900 | 183300 | 77.82 | 16.82 | 25.20 | 0.00 | 0.00 | 0.00 | 0.00 |
| 2015 | June | 627900 | 236100 | 52.70 | 7.99 | 11.30 | 0.00 | 0.83 | 0.00 | 0.83 |
| 2015 | June | 557300 | 234900 | 78.53 | 5.95 | 38.33 | 0.00 | 0.00 | 0.00 | 0.00 |
| 2015 | June | 349900 | 487200 | 55.45 | 0.00 | 0.84 | 0.00 | 0.00 | 0.00 | 0.00 |
| 2015 | June | 317200 | 669300 | 55.82 | 5.45 | 21.98 | 0.00 | 0.00 | 0.00 | 0.00 |
| 2015 | June | 626000 | 148000 | 78.62 | 18.35 | 28.50 | 0.00 | 0.38 | 1.61 | 1.99 |
| 2015 | June | 618200 | 247000 | 57.70 | 6.87 | 13.55 | 0.00 | 0.00 | 0.00 | 0.00 |
| 2015 | June | 452900 | 158900 | 60.01 | 1.36 | 10.72 | 0.00 | 1.37 | 0.00 | 1.37 |
| 2015 | June | 447700 | 173800 | 66.86 | 11.37 | 18.14 | 0.00 | 0.00 | 0.00 | 0.00 |
| 2015 | June | 623100 | 235100 | 66.67 | 1.74 | 16.43 | 0.00 | 0.00 | 0.00 | 0.00 |
| 2015 | June | 596400 | 316500 | 60.54 | 7.32 | 11.41 | 0.00 | 0.60 | 0.00 | 0.60 |
| 2015 | July | 527000 | 217100 | 61.66 | 1.41 | 22.47 | 0.38 | 0.00 | 0.00 | 0.38 |
| 2015 | July | 509000 | 268900 | 80.44 | 7.20 | 29.59 | 0.00 | 0.00 | 0.00 | 0.00 |
| 2015 | July | 622100 | 234600 | 63.77 | 2.58 | 14.71 | 0.00 | 0.00 | 0.00 | 0.00 |
| 2015 | July | 349000 | 105600 | 53.56 | 0.00 | 0.95 | 0.00 | 0.00 | 0.00 | 0.00 |
| 2015 | July | 447700 | 158300 | 76.58 | 10.93 | 27.44 | 0.00 | 0.56 | 0.53 | 1.09 |
| 2015 | July | 347800 | 368900 | 71.52 | 1.42 | 8.32 | 0.00 | 0.59 | 0.00 | 0.59 |
| 2015 | July | 589100 | 215300 | 55.54 | 4.41 | 7.67 | 0.00 | 0.00 | 0.00 | 0.00 |
| 2015 | July | 441700 | 157700 | 74.23 | 9.31 | 15.64 | 0.38 | 0.72 | 0.00 | 1.10 |
| 2015 | July | 454500 | 104600 | 46.41 | 3.66 | 10.59 | 0.00 | 0.00 | 0.38 | 0.38 |
| 2015 | July | 464600 | 107100 | 46.15 | 0.46 | 10.53 | 0.00 | 0.00 | 0.00 | 0.00 |
| 2015 | July | 606000 | 155000 | 51.36 | 0.53 | 11.97 | 0.00 | 0.65 | 0.00 | 0.65 |
| 2015 | July | 461600 | 191100 | 65.86 | 4.76 | 23.91 | 0.00 | 0.00 | 0.00 | 0.00 |
| 2015 | July | 437800 | 357200 | 50.67 | 0.00 | 3.50 | 0.00 | 0.00 | 0.00 | 0.00 |
| 2015 | July | 515500 | 172900 | 14.85 | 0.00 | 0.00 | 0.00 | 0.00 | 0.00 | 0.00 |
| 2015 | July | 430500 | 544100 | 49.86 | 2.55 | 10.89 | 0.00 | 0.00 | 0.00 | 0.00 |
| 2015 | July | 375500 | 166000 | 27.18 | 0.00 | 0.00 | 0.00 | 0.00 | 0.00 | 0.00 |
| 2015 | Aug. | 230700 | 335900 | 48.47 | 0.00 | 0.00 | 0.00 | 0.00 | 0.00 | 0.00 |
| 2015 | Aug. | 234200 | 619800 | 26.72 | 0.00 | 0.00 | 0.00 | 0.00 | 0.00 | 0.00 |
| 2015 | Aug. | 463700 | 176300 | 48.73 | 1.58 | 8.50 | 0.00 | 0.00 | 0.00 | 0.00 |
| 2015 | Aug. | 339600 | 94500 | 50.90 | 0.00 | 1.87 | 0.00 | 0.00 | 0.00 | 0.00 |
| 2015 | Aug. | 567900 | 205600 | 60.33 | 1.55 | 14.46 | 0.00 | 0.00 | 0.00 | 0.00 |
| 2015 | Aug. | 347000 | 93500 | 46.41 | 0.00 | 4.66 | 0.00 | 0.00 | 0.00 | 0.00 |
| 2015 | Aug. | 370700 | 253800 | 56.78 | 0.74 | 10.97 | 0.00 | 0.00 | 0.00 | 0.00 |
| 2015 | Aug. | 441900 | 177100 | 80.12 | 10.28 | 24.60 | 0.00 | 0.38 | 0.00 | 0.38 |
| 2015 | Aug. | 342400 | 275500 | 74.83 | 0.00 | 35.30 | 0.00 | 0.00 | 0.00 | 0.00 |
| 2015 | Aug. | 614100 | 158400 | 22.44 | 0.00 | 0.18 | 0.00 | 0.00 | 0.00 | 0.00 |
| 2015 | Aug. | 359800 | 181800 | 21.60 | 0.00 | 0.00 | 0.00 | 0.00 | 0.00 | 0.00 |
| 2015 | Aug. | 622600 | 254600 | 80.65 | 15.26 | 35.07 | 0.00 | 0.00 | 0.00 | 0.00 |
| 2015 | Aug. | 606200 | 136400 | 58.48 | 6.17 | 20.52 | 0.00 | 0.00 | 0.00 | 0.00 |
| 2015 | Aug. | 466800 | 183300 | 38.39 | 0.42 | 7.67 | 0.00 | 0.00 | 0.00 | 0.00 |
| 2015 | Aug. | 445800 | 164700 | 42.75 | 0.47 | 11.82 | 0.00 | 0.00 | 0.00 | 0.00 |
| 2015 | Aug. | 372400 | 167400 | 52.02 | 0.01 | 1.14 | 0.00 | 0.00 | 0.00 | 0.00 |
| 2015 | Aug. | 369900 | 180400 | 57.84 | 0.20 | 6.66 | 0.00 | 0.00 | 0.00 | 0.00 |
| 2015 | Aug. | 413500 | 297400 | 34.24 | 2.76 | 15.30 | 0.00 | 0.00 | 0.00 | 0.00 |
| 2015 | Aug. | 268300 | 232900 | 31.53 | 0.00 | 0.00 | 0.00 | 0.00 | 0.00 | 0.00 |
| 2015 | Aug. | 358900 | 175400 | 8.38 | 0.00 | 0.00 | 0.00 | 0.00 | 0.00 | 0.00 |
| 2015 | Aug. | 374400 | 163800 | 17.32 | 0.00 | 0.13 | 0.00 | 0.38 | 0.00 | 0.38 |
| 2015 | Aug. | 612800 | 235400 | 68.39 | 0.00 | 6.17 | 0.00 | 0.00 | 0.00 | 0.00 |
| 2015 | Aug. | 429800 | 546700 | 59.07 | 4.87 | 10.50 | 0.00 | 0.00 | 0.00 | 0.00 |
| 2015 | Aug. | 241500 | 663800 | 36.28 | 0.00 | 1.50 | 0.00 | 0.00 | 0.00 | 0.00 |
| 2015 | Aug. | 405300 | 245000 | 46.11 | 0.00 | 5.21 | 0.00 | 0.00 | 0.00 | 0.00 |
| 2015 | Aug. | 489100 | 144600 | 15.55 | 0.00 | 0.00 | 0.00 | 0.58 | 0.00 | 0.58 |
| 2015 | Aug. | 324800 | 671800 | 8.45 | 0.00 | 0.00 | 0.00 | 0.00 | 0.00 | 0.00 |
| 2015 | Aug. | 306000 | 529600 | 59.25 | 0.93 | 6.32 | 0.00 | 0.00 | 0.00 | 0.00 |
| 2015 | Aug. | 484600 | 145700 | 15.05 | 0.00 | 0.00 | 0.00 | 0.00 | 0.00 | 0.00 |
| 2015 | Aug. | 463600 | 176400 | 48.60 | 1.55 | 8.76 | 0.00 | 0.00 | 0.00 | 0.00 |
| 2015 | Aug. | 482800 | 149000 | 30.13 | 0.00 | 4.72 | 0.00 | 0.00 | 0.00 | 0.00 |
| 2015 | Aug. | 498600 | 126400 | 52.94 | 6.13 | 12.08 | 0.00 | 0.00 | 0.00 | 0.00 |
| 2015 | Aug. | 491600 | 144500 | 33.67 | 0.00 | 1.28 | 0.69 | 0.00 | 0.00 | 0.69 |
| 2015 | Aug. | 607400 | 230800 | 62.06 | 2.10 | 7.63 | 0.00 | 0.00 | 0.00 | 0.00 |
| 2015 | Aug. | 600700 | 227000 | 19.54 | 0.36 | 2.80 | 0.00 | 0.00 | 0.00 | 0.00 |
| 2015 | Aug. | 597400 | 234100 | 74.45 | 1.67 | 8.26 | 0.00 | 0.00 | 0.00 | 0.00 |
| 2015 | Aug. | 583400 | 186600 | 16.71 | 0.00 | 0.05 | 0.00 | 0.00 | 0.00 | 0.00 |
| 2015 | Aug. | 608900 | 237900 | 69.07 | 4.87 | 27.63 | 0.38 | 0.38 | 0.00 | 0.76 |
| 2015 | Aug. | 485500 | 138100 | 19.29 | 0.00 | 0.32 | 0.00 | 0.00 | 0.00 | 0.00 |
| 2015 | Aug. | 374600 | 113700 | 78.42 | 0.02 | 5.28 | 0.00 | 0.00 | 0.00 | 0.00 |
| 2015 | Aug. | 330400 | 96100 | 41.87 | 0.00 | 2.66 | 0.00 | 0.00 | 0.38 | 0.38 |
| 2015 | Aug. | 351300 | 291800 | 71.20 | 10.54 | 18.97 | 0.00 | 0.00 | 0.00 | 0.00 |
| 2015 | Aug. | 350500 | 138500 | 47.21 | 0.00 | 0.00 | 0.00 | 0.00 | 0.00 | 0.00 |
| 2015 | Aug. | 169300 | 29800 | 39.32 | 5.13 | 1.94 | 0.00 | 0.00 | 0.00 | 0.00 |
| 2015 | Aug. | 427400 | 572900 | 53.82 | 4.11 | 29.19 | 0.00 | 0.00 | 0.00 | 0.00 |
| 2015 | Aug. | 340800 | 94900 | 52.35 | 0.00 | 4.30 | 0.00 | 0.00 | 0.00 | 0.00 |
| 2015 | Sept. | 455200 | 174800 | 67.59 | 5.85 | 21.23 | 0.00 | 0.00 | 0.00 | 0.00 |
| 2015 | Sept. | 487800 | 137600 | 11.15 | 0.00 | 0.00 | 0.00 | 0.00 | 0.00 | 0.00 |
| 2015 | Sept. | 495500 | 145600 | 40.79 | 0.01 | 1.66 | 0.00 | 0.00 | 0.00 | 0.00 |
| 2015 | Sept. | 372700 | 189300 | 64.15 | 1.56 | 4.22 | 0.00 | 0.00 | 0.38 | 0.38 |
| 2015 | Sept. | 352500 | 377200 | 51.30 | 1.74 | 5.28 | 0.00 | 0.00 | 0.00 | 0.00 |
| 2015 | Sept. | 366400 | 1040600 | 37.62 | 0.00 | 2.06 | 0.00 | 0.00 | 0.00 | 0.00 |
| 2015 | Sept. | 491600 | 134600 | 22.19 | 0.00 | 3.22 | 0.00 | 0.00 | 0.00 | 0.00 |
| 2015 | Sept. | 318600 | 732000 | 77.20 | 5.68 | 13.65 | 0.00 | 0.00 | 0.00 | 0.00 |
| 2015 | Sept. | 488300 | 135700 | 4.60 | 0.00 | 0.00 | 0.00 | 0.00 | 0.00 | 0.00 |
| 2015 | Sept. | 379100 | 94400 | 81.00 | 7.38 | 14.67 | 0.00 | 0.00 | 0.00 | 0.00 |
| 2015 | Sept. | 404600 | 393800 | 20.78 | 0.00 | 0.19 | 0.00 | 0.00 | 0.00 | 0.00 |
| 2015 | Sept. | 428800 | 195600 | 70.37 | 2.67 | 7.21 | 0.00 | 0.00 | 0.00 | 0.00 |
| 2015 | Sept. | 352900 | 354500 | 61.92 | 0.00 | 0.77 | 0.00 | 0.00 | 0.00 | 0.00 |
| 2015 | Sept. | 458400 | 172400 | 52.78 | 0.00 | 7.60 | 0.00 | 0.00 | 0.00 | 0.00 |
| 2015 | Sept. | 619900 | 223800 | 69.47 | 6.95 | 38.48 | 0.00 | 0.00 | 0.00 | 0.00 |
| 2015 | Sept. | 636900 | 170900 | 16.62 | 0.00 | 1.78 | 0.00 | 0.00 | 0.00 | 0.00 |
| 2015 | Sept. | 577900 | 191800 | 62.02 | 12.76 | 9.31 | 0.00 | 0.00 | 0.00 | 0.00 |
| 2015 | Sept. | 361900 | 284700 | 69.66 | 4.72 | 19.75 | 0.00 | 0.00 | 0.00 | 0.00 |
| 2015 | Sept. | 597800 | 220300 | 45.73 | 0.91 | 4.57 | 0.00 | 0.00 | 0.00 | 0.00 |
| 2015 | Sept. | 482600 | 142500 | 23.17 | 0.00 | 2.00 | 0.00 | 0.00 | 0.00 | 0.00 |
| 2015 | Sept. | 425600 | 553600 | 51.72 | 1.36 | 21.44 | 0.00 | 0.00 | 0.00 | 0.00 |
| 2015 | Sept. | 493500 | 145300 | 44.78 | 0.00 | 3.11 | 0.00 | 0.00 | 0.00 | 0.00 |
| 2015 | Sept. | 270400 | 855100 | 37.43 | 6.41 | 2.16 | 0.00 | 0.00 | 0.00 | 0.00 |

**Table C.** **Pearson’s correlation coefficients between agricultural land use types.**  Summary correlations for 2015 data between the three land use types considered as fixed effect to explain neonicotinoid residues in honey. All land use cover was determined within a 2 km radius from hives where honey samples were removed.

|  | **Total arable** | **Winter cereals** | **Oilseed rape** |
| --- | --- | --- | --- |
| **Total arable** | 1.00 | 0.55 (*p<*0.01) | 0.62 (*p<*0.01) |
| **Winter cereal** | 0.55 (*p<*0.01) | 1.00 | 0.53 (*p<*0.01) |
| **Oilseed rape** | 0.62 (*p<*0.01) | 0.53 (*p<*0.01) | 1.00 |

**Table D.** **Likelihood ratio tests for honey residue responses to oilseed rape cover in the pre-moratorium year.** The significance of the response of combined neonicotinoid residues detected in honey (NNI = clothianidin + thiamethoxam + imidacloprid) in the year (2014) immediately preceding the effective European Union moratorium on the use of neonicotinoids. Combined neonicotinoids residues are correlated with potential agricultural sources of these pesticides in the form of oilseed rape cover (OSR), winter sown cereal cover and total arable cover. Likelihood Ratio Tests assess whether these responses explain more variance than intercept only model. Log Likelihood, number of parameters sample size (n) and *AICc* are also provided.

| **Model** | **Log Lik.** | **Para.** | **n** | ***AICc*** | **LRT** |
| --- | --- | --- | --- | --- | --- |
| µ = a_0_ + *a_1_**OSR | -16.00 | 4 | 21 | 42.50 | χ^2^_1_=5.62, p=0.02 |
| µ = a_0_ + *a_1_**Winter cereals | -18.26 | 4 | 21 | 47.02 | χ^2^_1_=1.10, p=0.29 |
| µ = a_0_ + *a_1_**Arable | -18.07 | 4 | 21 | 46.64 | χ^2^_1_=1.48, p=0.22 |
